# Optimizing an mHealth Intervention to Change Food Purchasing Behaviors for Cancer Prevention: Protocol for a Pilot Randomized Controlled Trial

**DOI:** 10.2196/39669

**Published:** 2022-06-24

**Authors:** Olivia Z Horgan, Nicole T Crane, Evan M Forman, Brandy-Joe Milliron, Nicole L Simone, Fengqing Zhang, Meghan L Butryn

**Affiliations:** 1 Center for Weight, Eating, and Lifestyle Science Department of Psychological and Brain Sciences Drexel University Philadelphia, PA United States; 2 Department of Nutrition Sciences Drexel University Philadelphia, PA United States; 3 Department of Radiation Oncology Sidney Kimmel Cancer Center Thomas Jefferson University Philadelphia, PA United States; 4 Department of Psychological and Brain Sciences Drexel University Philadelphia, PA United States

**Keywords:** mHealth, cancer prevention, grocery shopping, diet, eating, mobile phone

## Abstract

**Background:**

Dietary intake is a powerful modifiable factor that influences cancer risk; however, most US adults do not adhere to dietary guidelines for cancer prevention. One promising pathway for improving dietary adherence is targeting grocery shopping habits. Interventions might facilitate healthy grocery choices, with a combination of mHealth and traditional methods, by promoting the salience of dietary goals while shopping, enhancing motivation to make dietary changes, and increasing household support for healthy food purchasing.

**Objective:**

This pilot study will assess feasibility and acceptability of intervention components designed to improve adherence to dietary guidelines for cancer prevention (preliminary aim). The primary aim of the study is to quantify the effect of each intervention component, individually and in combination, on dietary intake (primary aim) and grocery store food purchases (exploratory aim). Mediation analyses will be conducted to understand the mechanisms of action (goal salience, motivation, and household support—secondary aims). The overarching goal is to optimize an mHealth intervention to be tested in a future fully powered clinical trial.

**Methods:**

The study enrolled adults (N=62) with low adherence to dietary recommendations for cancer prevention. In a 20-week program, all participants attend a nutrition education workshop and receive weekly educational messages through an app. A factorial design is used to test 4 intervention components: (1) location-triggered messages: educational messages are delivered when arriving at grocery stores; (2) reflections on the benefits of change: content is added to messages to encourage reflection on anticipated benefits of healthy eating, and participants attend an additional workshop session and 3 coach calls on this topic; (3) coach monitoring: food purchases are monitored digitally by a coach who sends personalized weekly app messages and conducts 3 coaching calls that focus on feedback about purchases; and (4) household support: another adult in the household receives messages designed to elicit support for healthy food purchasing, and support is addressed in 3 coach calls and an extra workshop session attended by the index participant and household member. Assessments are completed at weeks 0, 10, and 20 using self-report measures, as well as objective capture of grocery data from the point of purchase using store loyalty accounts.

**Results:**

The National Cancer Institute funded this study (R21CA252933) on July 7, 2020. Participant recruitment began in the spring of 2021 and concluded with the successful enrollment of 62 participants. Data collection is expected to be completed in the summer of 2022, and results are expected to be disseminated in the summer of 2023.

**Conclusions:**

The results of this study will inform the development of scalable interventions to lower cancer risk via changes in dietary intake.

**Trial Registration:**

ClinicalTrials.gov NCT04947150; https://clinicaltrials.gov/ct2/show/NCT04947150

**International Registered Report Identifier (IRRID):**

DERR1-10.2196/39669

## Introduction

### Background

In the United States, 1 out of 2 men and 1 out of 3 women will develop cancer during their lifetime [1]. Guidelines from leading organizations, including the American Cancer Society and the World Cancer Research Fund/American Institute for Cancer Research (WCRF/AICR), highlight diet as a key factor influencing cancer risk [2,3]. Adherence to dietary guidelines is associated with reduced risk of cancer incidence and cancer-related mortality [4-8]. Certain foods have properties that are protective against cancer (eg, fruits and vegetables [9,10]), whereas others have carcinogenic properties (eg, processed meat [11-13]). Diet is also the primary driver of obesity risk, which increases cancer risk [14,15]. At the population level, the effect of dietary intake on cancer risk is significant. For example, 20% to 60% of digestive tract cancers can be attributed to the low consumption of fruits and vegetables; pancreatic cancer risk increases by 22% for each additional 25 g/day of added sugar intake, and a 10% increase in the intake of ultraprocessed foods prospectively predicts a 12% increase in cancer risk [16-18]. Most Americans do not meet the WCRF/AICR dietary guidelines [19,20], with 60% of adults having inadequate fruit and vegetable intake [21], and nearly 90% consuming too much processed meat [22].

An efficient and scalable way to improve dietary adherence may be to focus intervention efforts on the decisions made while purchasing food for consumption at home. Across socioeconomic and racial groups, approximately 60% to 70% of calories consumed by US adults come from foods purchased in supermarkets and grocery stores [23-25], which are visited 1 to 2 times per week on average [26-28], including in urban areas [29,30]. Purchasing decisions that occur while grocery shopping each week have an outsized effect on dietary intake. Food cues in one’s environment strongly influence eating behavior [31-33]. Humans are biologically driven to have a hedonic response to foods that are high in salt, sugar, and fat; thus, if these foods are readily available in one’s home, self-regulation of dietary intake in the home will be challenging [23,34]. The types and amounts of foods available in individuals’ homes are strongly related to their dietary patterns, with the presence of unhealthy foods predicting greater calorie and fat intake and lower fruit and vegetable availability predicting lower consumption of these foods [35-40]. When healthy foods are purchased and brought into the home and unhealthy foods are not, minimal self-control is needed to make healthy eating choices at home, which should improve the overall dietary quality.

Making healthy decisions at the point of purchase is very challenging for several reasons: food decisions are often habitual, quick, and prioritize short-term perceived reward [41-43]; exposure to tempting food increases feelings of hunger and craving (likely driven by the dopamine system) [44]; and industry marketing fosters impulsive purchases of processed, palatable foods [45]. Previous interventions designed to change grocery shopping habits have primarily focused on providing dietary education but have produced only modest changes in food purchasing [46-48]. Other types of interventions in this area target the financial aspects of grocery shopping (eg, discounts and vouchers) and aspects of the grocery store environment (eg, item placement and advertising) to improve purchasing behavior [46]. These interventions show some initial efficacy, but widespread implementation of these approaches may not be feasible.

A review of the theory and literature in this area suggests that, to improve grocery shopping by enhancing self-regulation at the point of purchase, it may be necessary to target 3 key aspects involved in decision-making. First, goal salience is an underappreciated driver of eating behavior [49-52]. When individuals do not have nutrition-related goals in mind, food purchases are more likely to be influenced by factors such as familiarity, whereas reminding individuals of goals results in significantly healthier food purchases [50]. For example, adults in a grocery store who were primed with a healthy eating goal chose more minimally or nonprocessed foods and fewer ultraprocessed foods than those who did not receive a health goal reminder [53].

Second, the level of motivation to make healthy food choices is a key determinant of food purchasing decisions [54,55]. Reflecting on the anticipated benefits of healthy eating might facilitate dietary adherence by increasing motivation [56-63]. Supportive accountability is another factor that can enhance motivation and facilitate behavior change [64,65]. The presence of an observing other enhances accountability [66] by prompting self-evaluation and self-regulation [67-70], and positive feedback from an observer further enhances motivation [71,72].

Finally, social factors within one’s household, including support for healthy eating and the perceived food preferences of family members, exert a strong influence on food purchases [73-77]. Experimental studies have demonstrated that modifying social factors can improve food choice [77,78]. In summary, interventions may be more successful in improving healthy grocery store purchases to change the home food environment if they (1) promote goal salience at key moments of food purchase decision-making, (2) enhance motivation to make and sustain changes to the diet, and (3) increase household support and accountability for healthy food purchasing.

To maximize the potential for dissemination of this type of intervention, it is sensible for it to be delivered remotely (eg, via individual phone calls or group workshop sessions held via videoconferencing) and to incorporate mobile health (mHealth) technology, such as a smartphone app. mHealth allows for scalable interventions to be delivered in real-world contexts, in real time, including to low-income, rural, and older adult populations [79-84].

**Figure 1:**
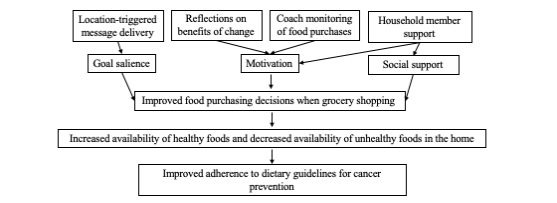
Project EatWell conceptual model.

This study tests 4 intervention components that target goal salience, motivation, and social support to facilitate food purchases that are consistent with cancer prevention dietary guidelines (Figure 1). The four intervention components are as follows:

1. Location-triggered messages: Educational messages are delivered via app just-in-time, that is, when individuals arrive at grocery shopping locations, to enhance goal salience. The mindfulness of program goals at the moment of decision-making is expected to facilitate program-consistent food purchasing behaviors.
2. Reflections on benefits of change: To enhance motivation, participants attend a 60-minute workshop and 3 coach calls to identify and reflect on the personal benefits of dietary change, and the content is added to educational app messages that prompt reflection on the anticipated rewards of healthy eating.
3. Coach monitoring: Food purchases are digitally monitored by a coach through a system that passively collects participants’ item-level data from stores, and the coach sends weekly app messages designed to enhance supportive accountability and thus motivation. Participants also attend 3 video calls to discuss their recent purchases with their coach.
4. Household support: Another adult in the household attends a 60-minute workshop and 3 coach calls with the index participant and receives weekly text messages designed to elicit support for healthy food purchasing and provide another source of supportive accountability for the index participant.

### Objectives of This Study

This pilot study uses a factorial design to test the effect of these 4 intervention components on grocery store purchases and adherence to the WCRF/AICR dietary recommendations for cancer prevention. The preliminary aim will assess feasibility and acceptability of the intervention components. The study also will quantify the effect of each intervention component individually and in combination on dietary intake (primary aim) and grocery store food purchases (exploratory aim). The secondary aim will use mediation analyses to explore whether changes in goal salience, motivation, and household social processes mediate differences in outcomes between conditions. Overall, the goal of the study is to inform future development and testing of interventions designed to change dietary intake.

## Methods

### Study Design

This study is a National Cancer Institute (NCI)-funded pilot, randomized controlled trial (R21CA252933) using a factorial design to test the effect of 4 different intervention components on dietary intake and grocery store purchases (Multimedia Appendix 1). The 4 factors yield 16 different combinations of intervention components (Table 1). An equal number of participants were randomized to receive versus not receive each of the 4 experimental intervention components. For example, half of the participants (31/62, 50%) have their food purchasing data monitored by a coach and half (31/62, 50%) do not have this feature as part of their intervention. As another example, half of the participants (31/62, 50%) were randomly assigned to have household member involvement included in their intervention package, but this randomization was done independently of that for coach monitoring (ie, the 4 experimental intervention components were not bundled together). The baseline covariates used for randomization included biological sex, BMI, age, household size, and dietary adherence score.

**Table 1:** Intervention components by condition.

Condition #	Condition	Workshops	Calls (all 20 minutes)	Messages per week
1	Control	Three 90-minute sessions	0	1 message (not location triggered)
2	LOC^a^	Three 90-minute sessions	0	1 message (location triggered)
3	BOC^b^	Three 90-minute sessions +one 60-minute BOC	3 BOC	1 message with BOC content (not location triggered)
4	HH^c^	Three 90-minute sessions +one 60-minute HH	3 HH	1 message (not location triggered) +1 HH text
5	CM^d^	Three 90-minute sessions	3 CM	1 message (not location triggered) +1 CM message
6	LOC+BOC	Three 90-minute sessions +one 60-minute BOC	3 BOC	1 message with BOC content (location triggered)
7	LOC+CM	Three 90-minute sessions	3 CM	1 message (location triggered) +1 CM message
8	LOC+HH	Three 90-minute sessions +one 60-minute HH	3 HH	1 message (location triggered) +1 HH text
9	BOC+HH	Three 90-minute sessions +one 60-minute BOC+one 60-minute HH	3BOC+3HH	1 message with BOC content (not location triggered) +1 HH text
10	BOC+CM	Three 90-minute sessions +one 60-minute BOC	3BOC+3CM	1 message with BOC content (not location triggered) +1 CM message
11	HH+CM	Three 90-minute sessions +one 60-minute HH	3 HH+ 3 CM	1 message (not location triggered) +1 CM message+1HH text message
12	LOC+HH+CM	Three 90-minute sessions +one 60-minute HH	3 HH+3 CM	1 message (location triggered) +1 CM message+1HH text
13	LOC+BOC+HH	Three 90-minute sessions +one 60-minute BOC+one 60-minute HH	3BOC+3HH	1 message with BOC content (location triggered) +1 HH text
14	LOC+BOC+CM	Three 90-minute sessions +one 60-minute BOC	3BOC+3CM	1 message with BOC content (location triggered) +1 CM message
15	BOC+HH+CM	Three 90-minute sessions +one 60-minute BOC +one 60-minute HH	3BOC+3HH+3CM	1 message with BOC content (not location triggered) +1 CM message+1HH text
16	LOC+BOC+HH+CM	Three 90-minute sessions +one 60-minute BOC+one 60-minute HH	3BOC+3 HH+3 CM	1 message with BOC content (location triggered) +1 CM message+1HH text message

^a^LOC: location-triggered messages.

^b^BOC: reflections on benefits of change.

^c^HH: household support.

^d^CM: coach monitoring.

### Ethics Approval

This study was approved by the Drexel University Institutional Review Board (study ID 2003007695) on March 13, 2021.

### Participants, Eligibility, and Recruitment

The study enrolled 62 index participants and 31 household members who served a support role. Participants were recruited from the Philadelphia area in 2 cohorts via targeted mailings, social media outreach, and Craigslist listings, and recruitment was supported in part by community recruitment resources from Thomas Jefferson University Sidney Kimmel Cancer Center. In particular, the Jefferson Regional Liaison Office used their honest broker system which aided in identifying and contacting potential participants using internal communication resources, community contacts, and other available resources (eg, participants within the Jefferson community who matched eligibility criteria were emailed about their interest in participating). Interested individuals completed a screening survey, and if deemed preliminarily eligible, attended an information session via videoconferencing. After the session, those interested in participating attended a baseline assessment to determine their final eligibility.

Index participants were required to be aged ≥18 years and fluent in English. In addition, participants were required to be the primary grocery shopper in their household and report shopping at stores that could passively stream item-level data from a store loyalty card to the study portal (Walmart, Target, ShopRite, or Wegmans). Inclusion criteria also included having a smartphone with an iOS or Android operating system that was compatible with the program app and living in a household with another adult who indicated willingness to participate in a support role. The exclusion criteria were as follows: medical condition or psychiatric condition (eg, active substance abuse or eating disorder) that would be a poor match with program content or limit ability to comply with program dietary recommendations, plans to enroll in another lifestyle modification program within 6 months of program start, bariatric surgery history, pregnancy or breastfeeding or plans to become pregnant in the next 6 months. All index participants provided written informed consent for participation, as did the 31 household members of the index participants randomized to receive the household support component.

### Intervention

#### Uniform Components

All index participants attend a nutrition education workshop (3 sessions of 90 minutes each, all delivered via videoconferencing) focused on eating a diet consistent with the WCRF/AICR guidelines. Content is organized around the key WCRF/AICR dietary recommendations: (1) eat a diet rich in whole grains, vegetables, and fruit; (2) limit consumption of highly processed foods; (3) limit consumption of red and processed meat; and (4) eliminate consumption of sugar-sweetened beverages. Sessions consist of psychoeducation about these nutrition recommendations, group discussions on health behavior change (eg, common triggers for eating behavior), didactics on behavioral skills (eg, stimulus control, functional analysis, and problem solving), and hands-on practice (eg, reading a nutrition label). Each workshop concludes with goal setting and meal planning, where participants identify concrete guideline-related goals for the coming week, create a weekly meal plan, and begin constructing a grocery list for relevant items. They are encouraged to complete their meal plan and grocery list independently after each session. The workshop sessions consist of 10 to 15 participants each. Coaches are experienced in delivering lifestyle modification and have a master’s degree or PhD in psychology, nutrition, or a related field. Each participant has continuity working with the same coach for all workshop sessions and, if applicable, any additional condition-specific contacts (ie, extra workshop sessions, coaching calls, and coach messages).

All index participants also download an app created for this program. A key feature of the app is the display of graphs that reflect how well the participant’s grocery shopping purchases align with each of the program recommendations across the previous 4 weeks. Participants are encouraged to use these graphs to track their progress and improvement toward recommendations over time. The grocery shopping data displayed in the graphs are passively collected from participant’s store loyalty accounts, as described in the *Grocery Store Purchases* section. During the 20-week intervention period, all participants also receive once-weekly educational messages in the app that remind them of program dietary recommendations and behavioral strategies that can promote adherence (eg, planning, self-monitoring, and goal setting). Message content includes tips for meeting program guidelines such as swapping out processed snacks for healthier alternatives or recipe ideas to incorporate fruits and vegetables (eg, “Replacing high-calorie, processed foods with fruits, vegetables, whole grains, beans, and legumes can help you feel fuller longer, have more energy, and better manage cravings and appetite, all of which can help you manage your weight. Identify one thing you could do this week to continue to make progress on the goal of replacing processed foods with healthier items”). These messages are standardized such that the content in any given week’s message is the same for all participants.

#### Experimental Intervention Components and Contact Time

##### Overview

As described next, the 4 experimental intervention components are each provided to 50% (31/62) of the participants, in addition to the 3 workshop sessions and standard weekly messages that all participants receive. The study was designed such that the program contact time varies by condition to evaluate the benefit of added contact time. The total number of workshop sessions ranges from 3 to 5, with 26% (16/62) of the participants assigned to 3 sessions (ie, no extra workshop sessions), 48% (30/62) assigned to 4 sessions (ie, an extra workshop session), and 26% (16/62) assigned to 5 sessions (ie, 2 extra workshop sessions). The total number of coach calls ranges from 0 to 9, with 18% (11/62) of the participants assigned to 0 calls, 29% (18/62) assigned to 3 calls, 39% (24/32) assigned to 6 calls, and 14% (9/62) assigned to 9 calls.

As described in detail in the next 4 component-specific sections, all participants receive a message from the app each week, which includes standardized educational content, and half of the participants (31/62, 50%) have benefits of change content appended to the message. For half of the participants (31/62, 50%), the delivery of the educational app message is location triggered. Half of the participants (31/62, 50%; ie, those who receive coach monitoring) also receive a second message in the app each week, written by their coach. Half of the participants (31/62, 50%) have a message sent to their household member each week.

##### Location-Triggered Messages

Participants randomized to receive this component receive their weekly educational message in the program app when their smartphone is within a 50-meter geofence around designated grocery stores. At baseline, participants provided information about the venues where they regularly grocery shop, for geofence programming. If the system does not detect the designated location in a given week (eg, the participant does not visit the grocery store), the app message is delivered at the end of the week. The message is delivered only once per week, even if the participant is at a grocery shopping location more than once.

Participants who are not assigned to location-triggered message delivery receive their weekly educational messages at a fixed time (ie, Sundays, at 10 AM), regardless of location. The content of the messages does not differ according to whether location-triggered messaging is provided.

##### Reflections on Benefits of Change

Participants randomized to receive the benefits of change component receive an extra 60-minute workshop session to reflect on the anticipated benefits of purchasing healthy food. They also attend 3 brief, individual coaching calls (20 minutes each) to further discuss personally meaningful benefits of change (at weeks 9, 13, and 17). Personalized content on their anticipated benefits is also added to each educational app message delivered after week 5. During the benefits of change workshop session, participants individually complete an exercise identifying short- and long-term benefits of healthy eating that are important to them, and message content is programmed according to the responses they record (eg, “Making healthy choices today will pay off in the long run because [I will be modeling these choices for my children, and they will benefit from healthier eating as well],” where bracketed input was generated by the participant).

Participants who do not receive the benefits of change component do not attend the additional workshop or these 3 coach calls focused on benefits of change and do not receive additional message content.

##### Coach Monitoring

For participants assigned to receive coach monitoring, the coach accesses a web-based portal where they view the participant’s food purchasing data, which are passively collected from the point of purchase using store loyalty accounts. The coach sends the participant a personalized message each week in the app, sharing feedback and observations from the food purchasing data. The participant also completes 3 calls with the coach (20 minutes each, held at weeks 4, 10, and 15) designed to further enhance supportive accountability for program goals. The coach messages and calls provide reinforcement for purchases consistent with program goals (particularly those that represent a change from baseline) and express concern for areas in which adherence is low.

If a participant is not assigned to receive coach monitoring, the coach has no objective information about food purchasing, and the participant does not receive any personalized coach messages in the app or phone calls focused on coach monitoring.

##### Household Support

Participants assigned to receive household support as part of the intervention select an adult in their household to serve in the support role. This household member receives weekly text messages (eg, “Your household member is likely trying to keep up new healthy habits for meal planning and grocery shopping. Identify one thing you can do to support their efforts with these changes this week. For instance, communicating in advance about meal and snack preferences, showing appreciation, or offering to look for healthy recipes to try”). In addition, the index participant and household member jointly participate in an extra 60-minute workshop session and three 20-minute coaching calls focused on household support (held at weeks 7, 11, and 16; the household member does not attend any other workshop sessions or coaching calls). The content of the workshops and calls is designed to elicit support for changing the home food environment and enhance supportive accountability by making household members aware of the index participant’s commitment to improving dietary intake.

For index participants who are not assigned to receive this intervention component, the household members have no program involvement.

### Measures

#### Feasibility and Acceptability

Feasibility and acceptability data are being collected and will be compared with preestablished benchmarks. Recruitment feasibility will be operationalized with a benchmark of >5 participants enrolled per month of recruitment and <30% of those otherwise interested and eligible refusing participation. Retention feasibility will be operationalized with a benchmark of >70% of the participants completing each follow-up assessment. Feasibility and acceptability of food purchasing data will be operationalized with a benchmark of >90% of the participants having their food purchase digital data captured successfully. Feasibility of message delivery will be assessed by location-triggered messaging delivery, with successful receipt of messages measured by <5% of deliveries encountering technological problems. User-rated acceptability will be measured using the benchmark of a mean rating >28 on the Treatment Acceptability Questionnaire (adapted, 8-items, 7-point Likert scale; given at 10 and 20 weeks) [85]. Qualitative information on acceptability will be collected via postintervention focus groups. Focus groups will be audio recorded; transcribed, with responses coded for themes and patterns; and used to further refine the intervention for future testing.

#### Dietary Intake

All participants complete dietary intake questionnaires at weeks 0 and 20. Cohort 1 participants completed 3 days of food recall at each time point, administered by the Automated Self-Administered 24-hour Dietary Recall (ASA24), an NCI-designed software tool [86]. ASA24 is based on the well-validated automated multiple pass method, which has been shown to be as or more accurate than nutritionist-administered 24-hour food recall when using doubly labeled water as the criterion [87,88].

After baseline administration of the ASA24, many cohort 1 participants reported that they perceived this measure to be excessively burdensome. Given its low acceptability, we replaced the ASA24 with the Diet History Questionnaire (DHQ-III) [89] for cohort 2 participants, chosen for its streamlined format and reduced completion time. The DHQ-III is a food frequency questionnaire developed by the NCI. The nutrient and food group database for the DHQ-III is based on a compilation of national 24-hour dietary recall data from the National Health and Nutrition Examination Surveys. Cohort 2 participants completed the DHQ-III at baseline and will complete it again at 20 weeks. Given the different measures of dietary intake, differences in dietary intake variables across waves will be assessed and analyses of dietary intake will be conducted separately for each wave.

Both the ASA24 and DHQ-III provide item-level nutritional information for all food and drinks consumed as well as daily totals of various nutrient variables [86,89]. For the NCI recommendation specific to highly processed food, food items will be flagged and included in this category based on those defined as highly processed according to the widely used NOVA classification system [90]. Items in the processed food category include salty snacks, frozen and prepared meals, baked goods, dessert, fried potatoes, candy, packaged bread and buns, refined grains, breakfast cereal, and processed cheese. Relevant items that fall into this category are pulled from the ASA24 item-level output based on their Food and Nutrient Database for Dietary Studies (FNDDS) food code [91] and from the DHQ-III item-level output based on their coding in the NCI’s associated nutrient database [92] and included in nutrient total calculations for processed foods. Similarly, sugar-sweetened drinks are identified in the ASA24 (based on their FNDDS food code) and DHQ-III (based on nutrient database) and used to calculate adherence to relevant guidelines. Sugar-sweetened drinks include nondiet sodas, nondiet fruit drinks, energy drinks, and sugary coffee drinks.

The average daily intake of the following items relevant to the NCI dietary recommendations will be calculated from the ASA24 and DHQ-III:

1. Fiber: grams of fiber
2. Fruit and vegetables: cups of all fruit (intact whole or cut fruit not including fruit juices) and vegetables (all vegetables excluding starches)
3. Added sugar from processed food: grams of added sugar consumed from items flagged as highly processed (as described earlier)
4. Saturated fat from processed food: grams of saturated fat consumed from items flagged as highly processed (as described earlier)
5. Sodium from processed food: milligrams of sodium consumed from items flagged as highly processed (as described earlier)
6. Red meat: ounces of beef, veal, pork, lamb, and game meat
7. Processed meat: grams of frankfurters, sausages, corned beef, and luncheon meat made from beef, pork, or poultry
8. Sugar-sweetened drinks: ounces of sugar-sweetened beverages (as defined earlier)

Given that NCI dietary recommendations for red and processed meat are at the weekly (vs daily) level, the average daily intake of red and processed meat is prorated to reflect intake over 7 days.

Scores for adherence in each domain of the NCI dietary recommendations are calculated based on the 0, 0.5, and 1 cutoff values established previously [93], where 1 reflects fully meeting the recommended level of intake, 0.5 indicates partially meeting recommended levels, and 0 reflects failure to meet the recommendation. When guidelines include multiple subcategories (eg, fiber and fruit or vegetables), the guideline score was calculated as the average of adherence to subcategories. Two overall adherence scores were calculated: (1) the sum of adherence to the 4 guidelines (range 0-4) and (2) average adherence to the 4 guidelines (range 0-1). The subscores are (1) average of adherence scores for fiber and fruit and vegetables; (2) average of adherence scores for added sugar, saturated fat, and sodium in processed foods; (3) average of adherence scores for red and processed meats; and (4) adherence score for sugar-sweetened drinks.

As a secondary outcome for dietary intake, an adapted, 13-item food frequency questionnaire [94-96] is administered at baseline and 20 weeks. Each item pertains to 1 of the 4 dietary guidelines (eg, “Whole grain products or high fiber starches,” “Red meats such as beef, pork, or lamb,” or “Nondiet sweet drinks”), and participants are instructed to report the frequency with which they ate the foods in the past month with a 6-point Likert-type scale (ranging from 0—“Never” to 5—“Twice or more per day”). Total guideline scores will be calculated as the average of responses to all items pertaining to that guideline (eg, guideline 1 as the average of 0-5 response for fiber and 0-5 response for fruit or vegetables).

#### Mediators

Consistent with the conceptual model of the study, we measured 3 potential mediators. An adapted Goal Salience Questionnaire is administered at each time point (baseline and 10 and 20 weeks) to measure dietary goal salience; that is, the extent to which participants think about dietary recommendations when grocery shopping [97]. Motivation for dietary adherence is measured at each time point using items adapted from the Treatment Self-Regulation Questionnaire [98]. A total of 2 measures of household social factors are administered at baseline, 10 weeks, and 20 weeks, where items were adapted to apply to one’s household rather than social network more broadly: the Supportive Accountability Questionnaire [71] and Sallis Social Support for Diet [99-101].

#### Moderators

Several potential moderators were measured at baseline. Participants completed a self-reported demographics questionnaire that gathered information about sex, race, ethnicity, age, education level, household size, and grocery shopping frequency. An adapted version of the Relationship Assessment Scale [102] was used to measure the quality of the relationship between the index participant and their household member. Weight, height, and weight history were measured using an investigator-developed weight-history questionnaire. Uncontrolled eating, cognitive restraint, and emotional eating were measured using a 21-item version of the Three Factor Eating Questionnaire-R21 [103] at baseline. The Three Factor Eating Questionnaire will also be completed after 10 and 20 weeks.

#### Household Member Information

Participating household members are administered the following questionnaires, which will be examined in exploratory analyses: an investigator-developed household demographics and goals questionnaire (at baseline only), the adapted food frequency questionnaire (same instrument administered to index participants, at baseline and 20 weeks), and the Treatment Acceptability Questionnaire (same instrument administered to index participants, at 20 weeks).

#### Grocery Store Purchases

Beginning 4 weeks before the intervention start date and continuing until 4 weeks after the intervention end date, study software, using an application programming interface (API), will continuously collect each participant’s item-level food purchases to objectively measure how grocery shopping changes over time. Participants provided store account credentials for loyalty programs at one or more of the four designated stores (Wegmans, Shop Rite, Target, and Walmart) to the study team at baseline. The API links item-level food purchases with nutrition databases (eg, FNDDS) to create summary nutrition variables, including added sugar, sodium, and saturated fat from processed foods for each item in a grocery trip. Change in purchase amounts in each nutrition category of interest related to the NCI guidelines (eg, ounces of sugar-sweetened beverages purchased per week) will be calculated. This will be an exploratory outcome because this method of data capture and categorization of grocery purchases is novel, and its feasibility has not yet been tested.

### Data Analysis

As a preliminary trial, analyses will focus on estimates of effect size. This study was not designed to be powered by statistical significance. The study is designed to provide information about feasibility, acceptability, and effect estimates (as well as CIs and estimates of variability) that will inform decisions about which components should be retained in the intervention package, with the goal of crafting a package that will produce an improvement in dietary intake of at least 10%, which is a criterion that corresponds to clinically meaningful changes in cancer risk [17,104]. The population-level impact of such a change would be meaningful.

For each of the 4 experimental intervention components, estimates of main effects will be calculated by comparing participants assigned to receive that component (n=31) to those that did not (n=31); thus, confidence in effect sizes is equivalent to that which would be achieved with a 2-arm trial with 62 participants. Analyses of covariance will be used to examine the effect of each experimental condition on the outcomes of interest, while controlling for the outcome variable at baseline. To test potential moderators of these relationships (eg, gender and race), analyses of covariance will examine the influence of experimental conditions, hypothesized moderators, and their interaction on the outcomes of interest, while controlling for the respective outcome variable baseline. To better understand the underlying mechanisms of these relationships, we will conduct mediation analysis using the Hayes PROCESS macro in SPSS (model 4, SPSS Inc). Models will examine whether temporally precedent changes in the proposed mediators (eg, goal salience, motivation, and household social processes) mediate differences in the primary outcomes between experimental conditions, while controlling for the outcome variable at baseline.

## Results

### Overview

**Figure 2:**
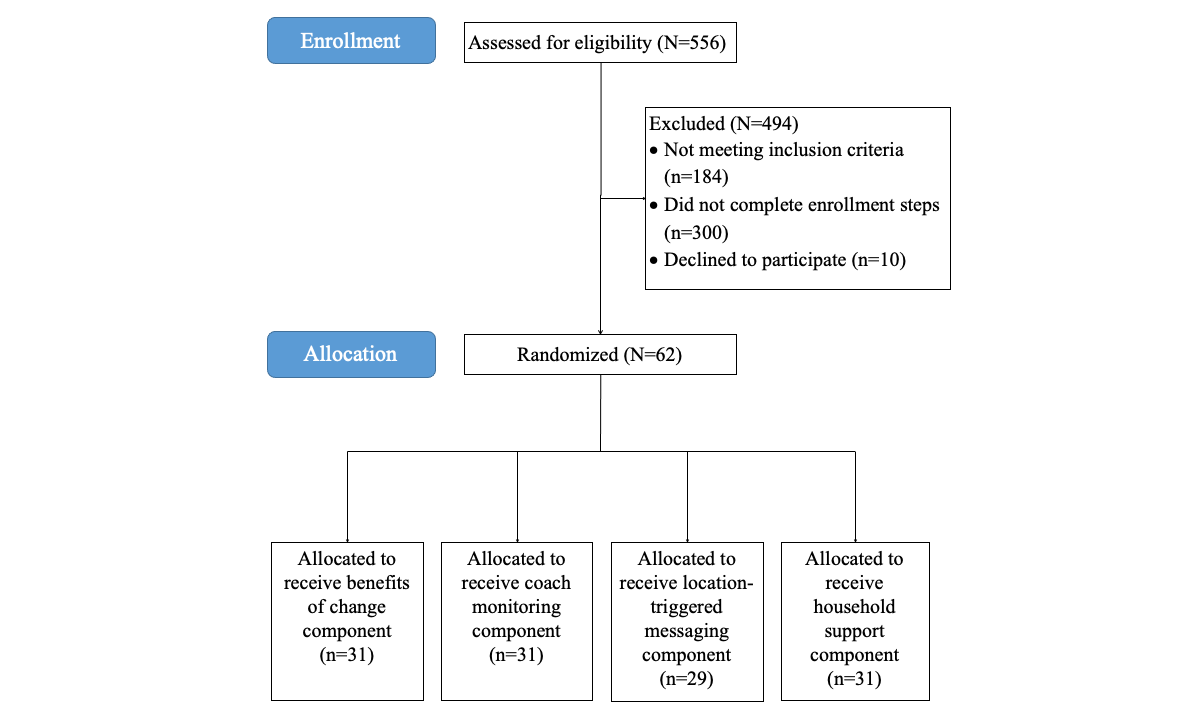
Project EatWell consort diagram.

The NCI funded this study (R21CA252933) on July 7, 2020, to be funded from July 1, 2020, to June 30, 2022. Participant recruitment was conducted in 2 distinct cohorts, beginning in spring 2021 and ending in fall 2021. In total, we screened 556 participants, 494 (88.8%) of which were excluded for (1) not meeting the inclusion criteria (184/556, 37.3%), (2) not completing subsequent enrollment steps (300/556, 60.7%), or (3) declining to participate (10/556, 2%). Following recruitment, 62 participants were enrolled, and each additional intervention component was randomly assigned to 50% (31/62) of the participants (Figure 2). As of April 2022, cohort 1 had completed their intervention period and 20-week assessments. Data collection for cohort 2 is expected to be completed in the summer of 2022, and results are expected to be disseminated in the summer of 2023.

### Baseline Characteristics

Participant demographic information was collected at baseline (Tables 2 and 3). The majority (57/62, 92%) of participants were female, and the mean age at baseline was 47.2 years (SD 13.5). Approximately half of the participants in the sample (32/62, 52%) are non-Hispanic White and 34% (21/62) are Black or African American. Most participants (47/62, 76%) have a college, graduate, or professional degree. Self-reported BMI at baseline was in the overweight or obese range for 80% (49/62) of the participants.

Descriptive statistics for dietary intake at baseline are presented in Table 4. These were calculated using data from the ASA24 in cohort 1 and DHQ-III in cohort 2. Average adherence scores were also calculated, where 1 reflects fully meeting the recommended level of intake, 0.5 indicates partially meeting the recommended levels, and 0 reflects failure to meet the recommendation. Mean adherence score for the 4 dietary guidelines targeted in this program was 0.39 in cohort 1 and 0.46 in cohort 2.

**Table 2:** Baseline demographic information (N=62).

Characteristics		Participants, n (%)
**Sex**		
	Female	57 (92)
	Male	5 (8)
**Ethnicity**		
	Hispanic or Latino	6 (10)
	Non-Hispanic or Latino	56 (90)
**Race**		
	American Indian or Alaska Native	1 (2)
	Asian	4 (7)
	Native Hawaiian or Other Pacific Islander	0 (0)
	Black or African American	21 (34)
	White	33 (53)
	More than one race	3 (5)
**Education level**		
	Completed senior high	3 (5)
	Completed some college	12 (19)
	Graduated from college	29 (47)
	Completed postgraduate or professional degree	18 (29)
**Household size (including index participant)**		
	2	24 (39)
	3	15 (24)
	4	16 (26)
	≥5	7 (11)
**Grocery shopping frequency**		
	Less than once per week	6 (10)
	Once per week	29 (47)
	Twice per week	18 (29)
	More than twice per week	9 (15)
**BMI range, kg/m^2^**		
	Underweight BMI (<18.5)	1 (2)
	Normal BMI (18.6-24.9)	12 (19)
	Overweight BMI (25-29.9)	17 (27)
	Obese BMI (>30)	32 (53)

**Table 3:** Baseline age and average body composition measurement.

	Mean (SD)	Range	
		Minimum	Maximum
Age (years)	47.2 (13.5)	23	69
Weight (kg)	85.6 (21.7)	42.6	135.6
BMI (kg/m^2^)	32.1 (8.0)	16.1	51.3

**Table 4:** Baseline dietary intake.

Category		Cohort 1, mean (SD)	Cohort 2, mean (SD)
**Daily averages**			
	Fiber (grams)	17.86 (7.06)	16.54 (8.63)
	Fruit and vegetables (cups)	2.02 (1.23)	2.67 (1.55)
	Added sugar in processed foods (grams)	38.43 (29.71)	26.22 (18.05)
	Saturated fat in processed foods (grams)	14.83 (8.54)	8.56 (4.51)
	Sodium in processed foods (milligrams)	1699.39 (842.84)	649.16 (315.19)
	Sugar-sweetened beverages (oz)	10.96 (10.57)	20.82 (29.88)
**Weekly averages**			
	Red meat (oz)	10.66 (15.48)	4.10 (3.12)
	Processed meat (grams)	228.28 (335.15)	149.30 (214.15)
**Adherence scores (range 0-1)**			
	Fiber, fruit, and vegetable guideline	0.39 (0.25)	0.43 (0.28)
	Processed foods guideline	0.38 (0.26)	0.61 (0.23)
	Red and processed meat guideline	0.50 (0.30)	0.56 (0.27)
	Sugar-sweetened beverages guideline	0.30 (0.34)	0.22 (0.31)
	Overall adherence (average)	0.39 (0.15)	0.46 (0.14)
	Overall adherence (sum)	1.57 (0.62)	1.85 (0.55)

## Discussion

### Principal Findings

Dietary intake is a critical modifiable risk factor of cancer. Grocery shopping is a potentially efficient and powerful intervention target; if individuals can make healthy purchases in stores, this will create optimal defaults in the home food environment that will make healthy eating more likely. However, in our modern obesogenic food environment, there is frequent exposure to tempting food cues, which makes healthy grocery store decisions difficult and demanding on self-regulatory capacity. Therefore, interventions to improve grocery shopping habits could focus on bolstering self-regulation at the key point of purchase.

The conceptual model proposed in this protocol attempts to improve grocery store purchases by targeting three key aspects of decision-making (goal salience, motivation, and social support) through four intervention components (location-triggered messages, benefits of change, coach monitoring, and household support) delivered via remotely delivered coaching and mHealth tools. The intervention moves beyond basic applications of stimulus control with appreciation for how challenging it is to change food purchasing habits. The tools used to promote behavior change are innovative, including geofence-triggered in-app messages to increase the salience of dietary goals and benefits of change at the moment of food purchasing, passive streaming of food purchase data to enable supportive accountability from a third party, and messaging and coaching to increase support and accountability at the household level.

Given the early stage of research on this type of intervention, methodical testing of the intervention components is needed. This study uses a factorial design to test the 4 intervention components and examine their feasibility, acceptability, and effect on food purchases and dietary intake, both individually and in combination. If the intervention components were tested at this stage in a 2-arm study (full package vs comparison condition) and found to be effective, it would be unknown which components contributed to the effect, how components influenced each other, or how to best make the intervention scalable and efficient [105].

### Strengths and Limitations

The tools used for the assessment and classification of food purchasing in this study are novel. The process of passively streaming item-level purchase data from the point of purchase to a database that can be used for both research (ie, outcome assessment) and clinical purposes (ie, coach monitoring) is a high-risk, high-reward element of the study. Successful demonstration of the use of this assessment tool would be a major contribution to the field’s efforts to create low-burden, high-validity options for collecting dietary data. Of course, although the objective nature of these food purchasing data is a strength, purchasing behavior does not align perfectly with dietary consumption. For example, individuals may purchase items at the grocery store that they do not eat themselves (eg, buying a snack item for their child) or that they only eat a small portion of (ie, share with others in their household). Food purchasing data also provide an incomplete picture of dietary intake, in that individuals may shop at food retailers outside of those accessible by our API system (eg, other grocery stores, farmers’ markets, or corner stores), and eat food items from other sources (eg, restaurants or social gatherings).

The use of geofencing to send location-triggered messages is also novel, as this technology has had only limited use in intervention studies. Using this new technology comes with the risk that the system may deliver messages at unintended times (ie, not when arriving at the grocery store) or fail to send them when expected, which would decrease the potency of the location-triggered text component of the intervention.

The protocol was launched during the COVID-19 pandemic and has had an impact on grocery shopping habits. When the pandemic began, studies show that the frequency of grocery shopping decreased to minimize exposure, and the types of foods purchased changed, driven more by what was available than by preference [106]. There were increases in the number of foods purchased during single grocery shopping trips, with people *stockpiling* out of fear of supply shortages [107]. Use of grocery delivery and pickup services has increased sharply since the pandemic began [108], showing increases of 158% and 255%, respectively [107]. However, at the same time, during the pandemic, individuals were also consuming less food outside the home [108], so in some ways grocery shopping may be an even better indicator of food intake than it previously was. Taken together, it may be more difficult to detect the effects of the intervention components, as change in purchases across the 20-week program could be influenced by pandemic-related confounds.

The self-reported assessment of dietary intake used in this study differs for cohort 1 and cohort 2 participants. Although the ASA24 (used in cohort 1) is a well-validated, frequently used measure with strong psychometric properties [109], participants reported that it had high burden and low acceptability. Therefore, the DHQ-III was used for cohort 2 participants. The DHQ-III is a traditional food frequency questionnaire, which has been cited in some studies as having less validity relative to the ASA24 [110], but it is a single-use, briefer questionnaire with lower burden, which may be necessary to maintain retention and engagement. Another weakness of the study’s assessment of dietary intake is that both the ASA24 and DHQ-III have limitations in their ability to capture all relevant food and drink items to quantify adherence to the WCRF/AICR guidelines. For example, in these types of dietary recalls, it can be difficult to differentiate between a processed can of soup that may contain high amounts of added sodium versus a homemade soup lower in sodium; however, these items have different implications in terms of adherence to guidelines for cancer risk.

### Conclusions

Despite these limitations, this study has the potential to advance the science of diet-related cancer prevention. This study is expected to lead to a large trial that will test an optimized package of intervention components. This trial will have the resources to test intervention effects for a longer period with a larger sample. A larger trial may also have the resources to incorporate additional assessment methods, such as objective measurement of the home food environment through home visits, use of ecological momentary assessment to illuminate decision-making while grocery shopping, and comprehensive assessment of household members’ dietary intake to measure the ripple effect of the intervention. If effective, these intervention efforts have the potential to meaningfully lower cancer risk at the population level. Importantly, although specific nutrition guidelines for cancer control may change in the future, this study’s contributions to the science of eating behavior change may be applied to various nutritional targets.

## Appendix

Multimedia Appendix 1 Original peer-review report from the National Institutes of Health for the grant proposal for this study.

## Abbreviations

AICR: American Institute for Cancer Research
API: application programming interface
ASA24: Automated Self-Administered 24-hour Dietary Recall
DHQ-III: Diet History Questionnaire-III
mHealth: mobile health
FNDDS: Food and Nutrient Database for Dietary Studies
NCI: National Cancer Institute
WCRF: World Cancer Research Fund

## Data Availability

The data generated during this study are available from the corresponding author upon reasonable request.
