# Supplementary material for: Optimizing an mHealth Intervention to Change Food Purchasing Behaviors for Cancer Prevention: Protocol for a Pilot Randomized Controlled Trial
Source: JMIR Res Protoc. 2022 Jun 24;11(6):e39669. doi: 10.2196/39669 (PMC9270710; doi:10.2196/39669)
Supplement: Multimedia Appendix 1 [file resprot_v11i6e39669_app1.pdf]

SUMMARY STATEMENT

PROGRAM CONTACT:
Tanya Agurs-Collins
240 276-6956
collinsta@mail.nih.gov

(Privileged Communication)

Release Date: **03/22/2020**
Revised Date:

Application Number: **1 R21 CA252933-01**

Principal Investigator

BUTRYN, MEGHAN

Applicant Organization: DREXEL UNIVERSITY

Review Group: **CLHP**
Community-Level Health Promotion Study Section

Meeting Date: **02/24/2020**
Council: **MAY 2020**
Requested Start: **07/01/2020**
RFA/PA: **PAR19-309**
PCC: **H1HP**

Project Title: **Optimizing an mHealth Intervention to Change Food Purchasing Behaviors for Cancer Prevention**
SRG Action: **Impact Score:31 Percentile:9 +**
Next Steps: **Visit https://grants.nih.gov/grants/next_steps.htm**
Human Subjects: 30-Human subjects involved - Certified, no SRG concerns
Animal Subjects: 10-No live vertebrate animals involved for competing appl.
Gender: 1A-Both genders, scientifically acceptable
Minority: 1A-Minorities and non-minorities, scientifically acceptable
Age: 3A-No children included, scientifically acceptable

Project Year	Direct Costs Requested	Estimated Total Cost
1	150,000	233,270
2	125,000	194,392
TOTAL	275,000	427,662

ADMINISTRATIVE BUDGET NOTE: The budget shown is the requested budget and has not been adjusted to reflect any recommendations made by reviewers. If an award is planned, the costs will be calculated by Institute grants management staff based on the recommendations outlined below in the COMMITTEE BUDGET RECOMMENDATIONS section.

1R21CA252933-01 Butryn, Meghan

RESUME AND SUMMARY OF DISCUSSION: This study aims to pilot test the effectiveness of four different mHealth intervention components on food purchasing behaviors using factorial design. Some reviewers thought the presented theoretical framework was interesting, while others felt that the rationale for the motivation aspect of the intervention needed to be better discussed. The investigative team is experienced in the proposed research area with a history of collaboration. The research environment is excellent. Other strengths of the proposed study include the use of the factorial design, the strong analytic plan, the use of the objective purchasing data, and the innovative use of the location-triggered text notifications. The effect of sex was taken into account in the randomization. Some weaknesses were identified during the discussion, including the possible attention bias and a lack of rationale for the dosage of text messages. Overall, however, the Committee agreed that this is a significant application from a strong research team. If successful, it will have a high public health impact.

DESCRIPTION (provided by applicant): Dietary intake is a powerful, modifiable factor that influences cancer risk. Unfortunately, most adults in the U.S. find it difficult to adhere to dietary guidelines for cancer prevention. One promising pathway for improving dietary adherence is to target grocery shopping habits, i.e., foods purchased for consumption at home. Two- thirds of daily food intake is sourced from or eaten in the home, so improving the quality of the home food environment should improve overall diet quality. When healthy foods are purchased and unhealthy foods are not, minimal self-control is needed to make healthy eating choices in the home. At the point of purchase, it is difficult to resist the temptation of palatable foods, but interventions might facilitate healthy choices by a) promoting dietary goal salience in real-time while grocery shopping, b) enhancing motivation to make and sustain changes to the diet, and c) increasing household support and accountability for healthy food purchasing. At this stage of research, methodical testing of intervention components that can change food purchase behaviors is needed, in order to craft an mHealth intervention package that is feasible, acceptable, and optimized for efficacy and scalability. The proposed study will enroll adults (N = 64) who have low adherence to cancer prevention dietary recommendations. All participants will attend a 3-hour nutrition education workshop. For 6 months following the workshop, all participants will receive text notifications that provide tailored reminders and recommendations for food purchasing. The study will use a factorial design to experimentally test four additional intervention components and examine their feasibility, acceptability, and effect on food purchases and dietary intake at 3 and 6 months. (Each component is randomly assigned to be activated for 50% of participants.) The four components to be tested are: 1) Location-triggered text notifications: Reminders and recommendations for food purchases are delivered “just-in-time,” when arriving at grocery shopping locations, to enhance goal salience. 2) Reflections on the benefits of change: To enhance motivation, content is added to messages to encourage reflection on the anticipated benefits of healthy eating. 3) Coach monitoring: Food purchases are automatically monitored by a coach (through a system that collects item-level store data) who sends personalized post-purchase messages designed to enhance supportive accountability and thus motivation. 4) Household text: Other adults in the household receive messages designed to elicit support for healthy food purchasing and provide another source of supportive accountability. The preliminary aim of the study is to assess feasibility and acceptability of the intervention components. The primary aim of the study is to quantify the effect of each intervention component, individually and in combination, on grocery store food purchases (objectively assessed with store data), and dietary intake (assessed with 24-hour food recalls). Mediation analyses also will be conducted. The overarching goal of this project is to optimize this mHealth intervention, which can be tested in the future in a fully powered clinical trial.

PUBLIC HEALTH RELEVANCE: Eating a healthy diet is important for cancer prevention. The foods that are purchased while grocery shopping strongly influence dietary intake. This study is designed to

evaluate how text notifications delivered to cell phones may change food purchasing habits and thus improve dietary intake.

CRITIQUE 1

Significance: 4
Investigator(s): 1
Innovation: 4
Approach: 3
Environment: 1

Overall Impact: This R21 application proposes to test the feasibility of four intervention components in a factorial experimental design. Each intervention component is delivered via smartphone and targets a theoretical construct related to dietary change. The four interventions are: 1) location-triggered texts to promote goal salience upon entering grocery stores, 2) texts to promote reflection on the benefits of dietary change to increase motivation, 3) texts from a coach based on passive grocery data monitoring providing feedback on purchases to increase supportive accountability, and finally 4) texts to another member of the household to increase support for the participant's healthy food purchases. The investigative team and environment are very strong. However, multiple weaknesses limit the potential of this study for public health impact. The rigor of prior research on motivation and mhealth dietary intervention is under-developed. The motivation measure also does not appear to match the way motivation is operationalized in the intervention. The goal salience location-triggered texts and the objective passive data streaming from loyalty cards are both highly innovative and potentially impactful areas for research. Unfortunately, the other proposed intervention components are much less compelling due to the weak rationale and unclear theoretical framework provided. The anticipated impact of the proposed study is moderate.

1. Significance

Strengths

- The investigators make a compelling case that education is necessary but not sufficient to produce meaningful dietary change.
- Grocery store food purchases are an appropriate target for intervention.
- The rationale for targeting goal salience is strong, and the investigators have preliminary data demonstrating the feasibility of methods for targeting this construct.
- Mhealth methods are well-suited to address grocery store food purchasing behavior.

Weaknesses

- The theoretical framework is confusing. Too little specific information is provided on the theory underlying each of the included constructs. The discussion of the rigor of prior research into each of these constructs is underdeveloped.
- The discussion of motivation, in particular, is shallow and should be clarified. Most of the works cited do not appear to use a Self-Determination Theory framework, and it is not clear which sub-constructs are being targeted specifically (Intrinsic motivation? Integrated regulation? Autonomous regulation overall?) The number of ACT interventions cited suggests that integrated regulation may be the most appropriate target, but this section as it is written does not provide sufficient rationale for the hypothesis that texts promoting reflection on benefits would increase a specific type of motivation which would then in turn impact eating behaviors.

- The rigor of prior mhealth studies is also not sufficiently covered. No case is made for the hypothesis that two texts per week is an appropriate dose of intervention or that texts relating to benefits, accountability, or social influence from other household members impact mediators or behavior.

2. Investigator(s)

Strengths

- Dr. Butryn is an outstanding investigator and very well-qualified to lead the proposed research study.
- The Co-Investigators and consultants are highly qualified with appropriate scopes of work matched to their areas of expertise.

Weaknesses

- None noted.

3. Innovation

Strengths

- Just-in-time location-based texting represents a very promising innovation for targeting grocery shopping behavior.
- Objective assessments of purchasing behavior will occur via a novel passive stream from loyalty cards.
- Use of a factorial design for feasibility studies is relatively rare.

Weaknesses

- A lack of adequate, specific explanation of the four theoretical constructs being targeted makes it difficult to determine the contribution to the literature.

4. Approach

Strengths

- The factorial design will allow simultaneous pilot testing of the feasibility of four different intervention components.
- Procedures for randomization are rigorous.
- Sex will be included as a variable in the algorithm for randomization.
- One intervention is assigned to each arm with clear differences between the type of texts participants in that group will receive.
- Specific benchmarks for major variables are discussed.
- The plan for mediation analysis is rigorous.
- Missing data will be dealt with appropriately.
- A passive, objective measure of grocery shopping will be the primary outcome, with the ASA24 serving as a standard measure of dietary intake.

Weaknesses

- It appears that participants in the location triggered text group will receive less attention (shorter/less frequent texts) than participants in some of the other groups. This difference in attention may bias results.
- The measure of motivation is from Self-Determination Theory and thus does not appear to match the way motivation is being operationalized in the intervention (e.g., referring to an amount of motivation, generally). It is not clear what types of motivation are the outcomes here an increase in autonomous, a decrease in controlled, etc.).
- Sex and several other variables are included in the randomization plan, but it is not clear whether they will be included in analyses as covariates.

5. Environment

Strengths

- The environment is excellent.

Weaknesses

- None noted.

Study Timeline

Strengths

- The timeline is very specific, with clear milestones.

Weaknesses

- None noted.

Protections for Human Subjects

Acceptable Risks and/or Adequate Protections

- Protections for human subjects are appropriate.

Data and Safety Monitoring Plan (Applicable for Clinical Trials Only):

Acceptable

- A DSMB will be convened. The data and safety monitoring plan is adequate.

Inclusion Plans

- Sex/Gender: Distribution justified scientifically
- Race/Ethnicity: Distribution justified scientifically
- For NIH-Defined Phase III trials, Plans for valid design and analysis: Not applicable
- Inclusion/Exclusion Based on Age: Distribution justified scientifically
- Men will be specifically targeted with several recruitment strategies. Children will not be included as they typically do not bear responsibility for grocery shopping. No upper age limit is included.

Vertebrate Animals

Not Applicable (No Vertebrate Animals)

Biohazards

Not Applicable (No Biohazards)

Authentication of Key Biological and/or Chemical Resources

Not Applicable (No Relevant Resources)

Budget and Period of Support

Recommend as Requested

- Budget appears appropriate.

CRITIQUE 2

Significance: 2
Investigator(s): 1
Innovation: 2
Approach: 3
Environment: 1

Overall Impact: This R21 project goal is to test a set of text message interventions to improve the healthfulness of grocery store purchases among 64 consumers with low adherence to cancer prevention dietary patterns. They will use a factorial design to test four interventions and examine their feasibility, acceptability, and effect on food purchases and dietary intake; they also aim to quantify the effect of each intervention component on purchases, using store data, and intake, using 24-hour recall. The long-term goal is to optimize these interventions in preparation for full scale trial. This is an experience PI and excellent team with a history of collaboration. There are minor weaknesses in significance and approach, but overall, this study has strong potential for impact in an important area and takes advantage of well conceptualized technology integration with behavior change.

1. Significance:

Strengths

- The importance of this area of work is justified.
- Prior research and gaps in the field is well articulated.
- The investigators’ own experience with a failed behavioral intervention and the follow up understanding they pursued is a notable strength.

Weaknesses

- There are data to suggest that online shopping is increasing dramatically, which is only minimally acknowledged.

2. Investigator(s):

Strengths

- This team is well qualified to conduct the research and has a strong history of collaboration.

Weaknesses

- None noted

3. Innovation:

Strengths

- Information Machine API for store level tracking.
- The factorial design is in this study context is exciting.
- The objective monitoring of purchases isn’t new but very appropriate for this study.

Weaknesses

- None noted

4. Approach:

Strengths

- Excellent descriptions of the interventions [4.2.c. Interventions] and outcomes [4.3. Outcome Measures], which are appropriate and comprehensive for this study
- A thoughtful, thorough statistical plan is provided
- Factorial design in this study context.

Weaknesses

- Could the initial 12 participants be in the intervention (n=64) study?
- There is a discrepancy with the planned enrollment, where it notes the 76 people, but then the intervention testing is happening with 64 (for which there is not planned enrollment table)
- Only those with smart phones can participate
- More information about access to the store data is needed.

5. Environment:

Strengths

- Excellent, supportive environments to carry out this project.

Weaknesses

- None noted

Study Timeline

Strengths

- The timeline is appropriate.

Weaknesses

- None noted

Protections for Human Subjects

Acceptable Risks and/or Adequate Protections

Data and Safety Monitoring Plan (Applicable for Clinical Trials Only):

Acceptable

Inclusion Plans

- Sex/Gender: Distribution justified scientifically
- Race/Ethnicity: Distribution justified scientifically
- For NIH-Defined Phase III trials, Plans for valid design and analysis: Not applicable
- Inclusion/Exclusion Based on Age: Distribution justified scientifically

Vertebrate Animals
Not Applicable (No Vertebrate Animals)

Biohazards
Not Applicable (No Biohazards)

Resource Sharing Plans
Acceptable

Authentication of Key Biological and/or Chemical Resources
Not Applicable (No Relevant Resources)

Budget and Period of Support
Recommend as Requested

CRITIQUE 3

Significance: 2
Investigator(s): 2
Innovation: 1
Approach: 2
Environment: 2

Overall Impact: This study will use a factorial design to experimentally test four intervention components and examine their feasibility, acceptability, and effect on food purchases and dietary intake at 3 and 6 months. (Each component is randomly assigned to be activated for 50% of participants.) The four components to be tested are: 1) Location-triggered text notifications: Reminders and recommendations for food purchases are delivered "just-in-time," when arriving at grocery shopping locations, to enhance goal salience. 2) Reflections on the benefits of change: To enhance motivation, content is added to messages to encourage reflection on the anticipated benefits of healthy eating. 3) Coach monitoring: Food purchases are automatically monitored by a coach (through a system that collects item-level store data) who sends personalized post-purchase messages designed to enhance supportive accountability and thus motivation. 4) Household text: Other adults in the household receive messages designed to elicit support for healthy food purchasing and provide another source of supportive accountability. The preliminary aim of the study is to assess feasibility and acceptability of

the intervention components. The primary aim of the study is to quantify the effect of each intervention component, individually and in combination, on grocery store food purchases (objectively assessed with store data), and dietary intake (assessed with 24-hour food recalls). Mediation analyses also will be conducted. The investigators propose to develop and test the feasibility of a new mHealth app to influence food purchasing practices. The goal is novel and the conceptual foundation and intervention procedure is innovative. The team is expert in related development activities, but has minimal involvement/expertise in home food purchasing behaviors. Rigor of Prior Research: The investigators reviewed some of the literature on food purchasing behavior and home food environment. They did not identify the strengths and weaknesses of this research, or what holes they will plug with their research.

1. Significance:

Strengths

- The investigators proposed a seemingly comprehensive four component approach to influence healthy food purchasing.

Weaknesses

- None noted

2. Investigator(s):

Strengths

- Dr. Butryn has experience with several mHealth interventions, but doesn't spell these out in her Biosketch.
- Dr. Forman has substantial experience in the design and evaluation of JITAI interventions.
- Dr. Milliron lists two publications on food purchasing, and is expert in the use of qualitative methods in the design of interventions.
- The team has expertise in most aspects of the proposed research.

Weaknesses

- The PI reports one involvement in research involving food purchasing. It would have been helpful for the PI to have more expertise in food purchasing behavior.
- It is not clear why so many faculty and consultants with overlapping expertise are involved in this R21 project.
- Although Dr. Milliron lists expertise in qualitative research to facilitate the design of interventions, no such qualitative research was proposed.

3. Innovation:

Strengths

- Innovations in the proposed research includes the conceptual model, the tools used to promote behavior change and the assessment of food purchasing.

Weaknesses

- None noted

4. Approach:

Strengths

- The investigators propose to develop and test the feasibility of a new mHealth app to influence food purchasing practices. The goal is novel and the conceptual foundation and intervention procedure is innovative.
- An interesting theoretical framework undergirds the proposed research.
- Rigor of Prior Research
- The investigators reviewed some of the literature on food purchasing behavior and home food environment. They did not identify the strengths and weaknesses of this research, or what holes they will plug with their research.
- Inclusionary criteria specify participants will be 18 years of age or older. Since people this age are likely to be the main home food purchaser, this age makes sense. Alternatively, 18+ years encompasses a broad variety of developmental stages with differing a) food intake patterns, b) likely food purchasing patterns, c) use of media, preference for type of media communications, etc. This was not discussed in the application.

Weaknesses

- The investigators propose extensive statistical analysis plans, but report no power calculations.
- Given the sample size and four factors in the design, the study will be underpowered to test effect individually or in combination, or to assess mediation. There are accepted standards for reporting feasibility studies. These guidelines should be followed. See S Eldridge et al. BMJ 2016, 355:i5239.
- The intervention specifies a 3-hour workshop to learn about dietary guidelines for cancer prevention. This will be a barrier to any approach to scaling up the intervention. Can abbreviated versions be offered on the internet in small pieces?
- It would appear that the location triggered text notifications (LTTN) would be more effective than generic messages if the LTTN were tailored to the participant's usual purchases at a store?
- The Household Text intervention appears to assume that families have another adult in the home, and that the relationship between members is of equality and openness to new information. The home relationship can vary substantially. It is common in Hispanic families for adult males to be the major influence on food purchasing, but the adult female responsible for preparation. It might be helpful for the investigators to employ some family relationship tool assessment baseline with household texts tailored to the relationship.
- The proposed mediating variables will not be validated for use in the proposed circumstances. No validity data were provided from comparable samples under comparable circumstances.

5. Environment:

Strengths

- Drexel University has recently been recognized as a research level university which can support the proposed research.

Weaknesses

- None noted

Protections for Human Subjects

Acceptable Risks and/or Adequate Protections

- No concerns.

Data and Safety Monitoring Plan (Applicable for Clinical Trials Only):

Acceptable

- No concerns.

Inclusion Plans

- Sex/Gender: Distribution justified scientifically
- Race/Ethnicity: Distribution justified scientifically
- For NIH-Defined Phase III trials, Plans for valid design and analysis: Scientifically acceptable
- Inclusion/Exclusion Based on Age: Distribution justified scientifically
- No concerns.

Vertebrate Animals

Not Applicable (No Vertebrate Animals)

Biohazards

Not Applicable (No Biohazards)

Resource Sharing Plans

Not Applicable (No Relevant Resources)

Authentication of Key Biological and/or Chemical Resources

Not Applicable (No Relevant Resources)

Budget and Period of Support

Recommend as Requested

THE FOLLOWING SECTIONS WERE PREPARED BY THE SCIENTIFIC REVIEW OFFICER TO SUMMARIZE THE OUTCOME OF DISCUSSIONS OF THE REVIEW COMMITTEE, OR REVIEWERS' WRITTEN CRITIQUES, ON THE FOLLOWING ISSUES:

PROTECTION OF HUMAN SUBJECTS: ACCEPTABLE

INCLUSION OF WOMEN PLAN: ACCEPTABLE

INCLUSION OF MINORITIES PLAN: ACCEPTABLE

INCLUSION ACROSS THE LIFESPAN: ACCEPTABLE

COMMITTEE BUDGET RECOMMENDATIONS: The budget was recommended as requested.

Footnotes for 1 R21 CA252933-01; PI Name: Butryn, Meghan

+ Derived from the range of percentile values calculated for the study section that reviewed this application.

NIH has modified its policy regarding the receipt of resubmissions (amended applications).See Guide Notice NOT-OD-18-197 at https://grants.nih.gov/grants/guide/notice-files/NOT-OD-18-197.html. The impact/priority score is calculated after discussion of an application by averaging the overall scores (1-9) given by all voting reviewers on the committee and multiplying by 10. The criterion scores are submitted prior to the meeting by the individual reviewers assigned to an application, and are not discussed specifically at the review meeting or calculated into the overall impact score. Some applications also receive a percentile ranking. For details on the review process, see http://grants.nih.gov/grants/peer_review_process.htm#scoring.

MEETING ROSTER

Community-Level Health Promotion Study Section
Healthcare Delivery and Methodologies Integrated Review Group
CENTER FOR SCIENTIFIC REVIEW
CLHP
02/24/2020 - 02/25/2020

Notice of NIH Policy to All Applicants: Meeting rosters are provided for information purposes only. Applicant investigators and institutional officials must not communicate directly with study section members about an application before or after the review. Failure to observe this policy will create a serious breach of integrity in the peer review process, and may lead to actions outlined in NOT-OD-14-073 at https://grants.nih.gov/grants/guide/notice-files/NOT-OD-14-073.html and NOT-OD-15-106 at https://grants.nih.gov/grants/guide/notice-files/NOT-OD-15-106.html, including removal of the application from immediate review.

CHAIRPERSON(S)

WARD, DIANNE STANTON, EDD
PROFESSOR
DEPARTMENT OF NUTRITION
GILLINGS SCHOOL OF GLOBAL PUBLIC HEALTH
UNIVERSITY OF NORTH CAROLINA AT CHAPEL HILL
CHAPEL HILL, NC 27599

MEMBERS

AFFUSO, HELEN OLIVIA, PHD
ASSOCIATE PROFESSOR
DEPARTMENT OF EPIDEMOLOGY
UNIVERSITY OF ALABAMA AT BIRMINGHAM
BIRMINGHAM, AL 35294

ALEXEEFF, STACEY E, BS, MS, PHD *
RESEARCH SCIENTIST I BIOSTATISTICIAN
DEPARTMENT OF RESEARCH
KAISER PERMANENTE NORTHERN CALIFORNIA
OAKLAND, CA 94612

ARREDONDO, ELVA M, PHD
PROFESSOR
DEPARTMENT OF HEALTH PROMOTION AND
BEHAVIORAL SCIENCE
SAN DIEGO STATE UNIVERSITY
SAN DIEGO, CA 92123

BARANOWSKI, TOM, PHD
DISTINGUISHED EMERITUS PROFESSOR
DEPARTMENT OF PEDIATRICS
CHILDREN'S NUTRITION RESEARCH CENTER
BAYLOR COLLEGE OF MEDICINE
HOUSTON, TX 77030

BERG, CARLA J, PHD
PROFESSOR
DEPARTMENT OF PREVENTION AND COMMUNITY HEALTH
GEORGE WASHINGTON UNIVERSITY
WASHINGTON, DC 20052

CHANG, TAMMY, MD *
ASSISTANT PROFESSOR
DEPARTMENT OF FAMILY MEDICINE
UNIVERSITY OF MICHIGAN AT ANN ARBOR
ANN ARBOR, MI 48105

CHOI, WON S, MPH, PHD
PROFESSOR
DEPARTMENT OF POPULATION HEALTH
SCHOOL OF MEDICINE
UNIVERSITY OF KANSAS MEDICAL CENTER
KANSAS CITY, KS 66160

COHEN, DEBORAH A, MD, MPH
RESEARCH SCIENTIST III
DEPARTMENT OF RESEARCH AND EVALUATION
KAISER PERMANENTE SOUTHERN CALIFORNIA
PASADENA, CA 91101

DAMASHEK, AMY L, MS, BS, PHD *
ASSOCIATE PROFESSOR
DEPARTMENT OF PSYCHOLOGY
WESTERN MICHIGAN UNIVERSITY
KALAMAZOO, MI 49008

DANILOVICH, MARGARET K, BA, DPT, PHD *
RESEARCH SCIENTIST AND DIRECTOR
LEONARD SCHANFIELD RESEARCH INSTITUTE
CJE SENIORLIFE
CHICAGO, IL 60201

DAVIS, KELLY CUE, PHD, MS *
ASSOCIATE PROFESSOR
EDSON COLLEGE OF NURSING AND HEALTH INNOVATION
CENTER FOR HEALTH PROMOTION AND DISEASE
PREVENTION
ARIZONA STATE UNIVERSITY
PHOENIX, AZ 98105

FEDERMAN, ALEX D, MD, MPH
PROFESSOR
DEPARTMENT OF GENERAL INTERNAL MEDICINE
ICAHN SCHOOL OF MEDICINE AT MOUNT SINAI
NEW YORK, NY 10029

FREEDMAN, DARCY ANN, MPH, PHD
PROFESSOR
DIRECTOR, SWETLAND CENTER FOR ENVIRONMENTAL HEALTH
DEPARTMENT OF POPULATION AND QUANTITATIVE HEALTH SCIENCES
CASE WESTERN RESERVE UNIVERSITY SCHOOL OF MEDICINE
CLEVELAND, OH 44106

GITTELSOHN, JOEL, PHD
PROFESSOR
DEPARTMENT OF INTERNATIONAL HEALTH
CENTER FOR HUMAN NUTRITION
BLOOMBERG SCHOOL OF PUBLIC HEALTH
JOHNS HOPKINS UNIVERSITY
BALTIMORE, MD 21205

HECKMAN, CAROLYN J, PHD
ASSOCIATE PROFESSOR
DEPARTMENT OF MEDICAL ONCOLOGY,
SECTION OF POPULATION SCIENCE
ROBERT WOOD JOHNSON MEDICAL SCHOOL
RUTGERS, THE STATE UNIVERSITY OF NEW JERSEY
NEW BRUNSWICK, NJ 08901

HUH, JIMI, BA, MA, PHD *
ASSOCIATE PROFESSOR
DEPARTMENT OF PREVENTIVE MEDICINE
KECK SCHOOL OF MEDICINE
UNIVERISTY OF SOUTHERN CALIFORNIA
LOS ANGELES, CA 90033-3628

JERNIGAN, VALARIE J, DRPH, MPH
PROFESSOR
DIRECTOR, CENTER FOR INDIGENOUS HEALTH RESEARCH AND POLICY
OKLAHOMA STATE UNIVERSITY
CENTER FOR HEALTH SCIENCES
TULSA, OK 74106

KATZ, MIRA L, PHD, MPH *
PROFESSOR
DEPARTMENT OF HEALTH BEHAVIOR
AND HEALTH PROMOTION
COLLEGE OF PUBLIC HEALTH
OHIO STATE UNIVERSITY
COLUMBUS, OH 43210

KOINIS MITCHELL, DAPHNE, PHD
PROFESSOR
DEPARTMENT OF PSYCHIATRY AND HUMAN BEHAVIOR AND
DEPARTMENT OF PEDIATRICS
BROWN MEDICAL SCHOOL
PROVIDENCE, RI 02903

LEE, HEE YUN, PHD *
PROFESSOR AND ASSOCIATE DEAN FOR RESEARCH
SCHOOL OF SOCIAL WORK
UNIVERSITY OF ALABAMA
TUSCALOOSA, AL 35401

LYNCH, ELIZABETH B, PHD *
ASSOCIATE PROFESSOR
DEPARTMENT OF PREVENTIVE MEDICINE
RUSH UNIVERSITY MEDICAL CENTER
CHICAGO, IL 60612

LYONS, ELIZABETH J., BA, MPH, PHD *
ASSOCIATE PROFESSOR
DEPARTMENT OF NUTRITION AND METABOLISM
THE UNIVERSITY OF TEXAS MEDICAL BRANCH
GALVESTON, TX 77550

MOON, RACHEL Y, MD *
PROFESSOR
DEPARTMENT OF PEDIATRICS
SCHOOL OF MEDICINE
UNIVERSITY OF VIRGINIA
CHARLOTTESVILLE, VA 22908

NICCOLAI, LINDA M, PHD
PROFESSOR
DEPARTMENT OF EPIDEMIOLOGY OF MICROBIAL DISEASES
YALE SCHOOL OF PUBLIC HEALTH
NEW HAVEN, CT 06520

SEGUIN-FOWLER, REBECCA ANNE, BS, MS, PHD *
ASSOCIATE PROFESSOR
DEPARTMENT OF NUTRITION
COLLEGE OF AGRICULTURE AND LIFE SCIENCES
TEXAS A&M UNIVERISTY
COLLEGE STATION, TX 77843

SMITH, GREGORY C, PHD
PROFESSOR
DIRECTOR, HUMAN DEVELOPMENT CENTER
LIFESPAN DEVELOPMENT AND EDUCATIONAL SCIENCE
COLLEGE OF EDUCATION, HEALTH AND HUMAN SERVICES
KENT STATE UNIVERSITY
KENT, OH 44242

SORKIN, DARA H, PHD
ASSOCIATE PROFESSOR
DIVISION OF GENERAL INTERNAL MEDICINE
UNIVERSITY OF CALIFORNIA, IRVINE
IRVINE, CA 92697

SWARTZ, JAMES ANTHONY, BA, MA, PHD *
PROFESSOR
JANE ADDAMS COLLEGE OF SOCIAL WORK
UNIVERSITY OF ILLINOIS AT CHICAGO
CHICAGO, IL 60607

WANG, MAY C, DRPH, MPH
PROFESSOR
DEPARTMENT OF COMMUNITY HEALTH SCIENCES
FIELDING SCHOOL OF PUBLIC HEALTH
UNIVERSITY OF CALIFORNIA LOS ANGELES
LOS ANGELES, CA 90095

WEYBRIGHT, ELIZABETH H, BS, MS, PHD *
ASSISTANT PROFESSOR
DEPARTMENT OF HUMAN DEVELOPMENT
WASHINGTON STATE UNIVERSITY
PULLMAN, WA 99164-4852

WU, LI-TZY T, SCD
PROFESSOR
DEPARTMENT OF PSYCHIATRY
SCHOOL OF MEDICINE
DUKE UNIVERSITY MEDICAL CENTER
DURHAM, NC 27710

YOUNG, HENRY N, PHD *
KROGER ASSOCIATE PROFESSOR
DEPARTMENT OF CLINICAL AND ADMINISTRATIVE PHARMACY
UNIVERSITY OF GEORGIA
ATHENS, GA 30602

MAIL REVIEWER(S)

DOMBROWSKI, KIRK, PHD, MA, AB
JOHN BRUHN PROFESSOR OF SOCIOLOGY
DEPARTMENT OF SOCIOLOGY
COLLEGE OF ARTS AND SCIENCES
UNIVERSITY OF NEBRASKA-LINCOLN
LINCOLN, NE 68588

IZURIETA, RICARDO OSWALDO, DPH, MD, MPH
ASSOCIATE PROFESSOR
DEPARTMENT OF GLOBAL HEALTH
COLLEGE OF PUBLIC HEALTH
UNIVERSITY OF SOUTH FLORIDA
TAMPA, FL 33612

SCHLADER, ZACHARY JOSEPH, PHD, MS, BS
ASSOCIATE PROFESSOR
DEPARTMENT OF KINESIOLOGY
INDIANA UNIVERSITY BLOOMINGTON
BLOOMINGTON, IN 14214

ZHANG, GQ, PHD, MS, BS
VICE PRESIDENT AND PROFESSOR
CHIEF OF DATA SCIENTIST
DEPARTMENT OF NEUROLOGY
MCGOVERN MEDICAL SCHOOL
UNIVERSITY OF TEXAS HEALTH SCIENCE CENTER, HOUSTON
HOUSTON, TX 77030

SCIENTIFIC REVIEW OFFICER

WU, PING, PHD
SCIENTIFIC REVIEW OFFICER
CENTER FOR SCIENTIFIC REVIEW
NATIONAL INSTITUTES OF HEALTH
BETHESDA, MD 20892

EXTRAMURAL SUPPORT ASSISTANT

NJOKU, PHILIP C
EXTRAMURAL SUPPORT ASSISTANT
DIVISION OF AIDS, BEHAVORIAL, POPULATION SCIENCES HEALTHCARE DELIVERY AND METHODOLOGIES (HDM)
NATIONAL INSTITUTES OF HEALTH
BETHESDA, MD 20892

* Temporary Member. For grant applications, temporary members may participate in the entire meeting or may review only selected applications as needed.

Consultants are required to absent themselves from the room during the review of any application if their presence would constitute or appear to constitute a conflict of interest.
